# Autonomous Collision Avoidance at Sea: A Survey

**DOI:** 10.3389/frobt.2021.739013

**Published:** 2021-09-16

**Authors:** Hans-Christoph Burmeister, Manfred Constapel

**Affiliations:** Fraunhofer Center for Maritime Logistics and Services, Hamburg, Germany

**Keywords:** COLREGs, collision avoidance(CA), maritime autonomous surface ship (MASS), autonomous surface vehicles(ASV), autonomous navigation, autonomous decision-making

## Abstract

In this survey, results from an investigation on collision avoidance and path planning methods developed in recent research are provided. In particular, existing methods based on Artificial Intelligence, data-driven methods based on Machine Learning, and other Data Science approaches are investigated to provide a comprehensive overview of maritime collision avoidance techniques applicable to Maritime Autonomous Surface Ships. Relevant aspects of those methods and approaches are summarized and put into suitable perspectives. As autonomous systems are expected to operate alongside or in place of conventionally manned vessels, they must comply with the COLREGs for robust decision-support/-making. Thus, the survey specifically covers how COLREGs are addressed by the investigated methods and approaches. A conclusion regarding their utilization in industrial implementations is drawn.

## 1 Introduction

Automation technologies, decision support, and autonomous navigation systems are becoming increasingly prevalent in shipping. These developments are driven by the core requirement to ensure safe and efficient operation and navigation of ships. In addition to avoiding groundings and stable navigation through rough weather conditions in the deep sea, collision avoidance is the main task for automated or autonomous navigation systems (Burmeister et al. (2020)). Safe and automated decision-making will thus become a necessity on Maritime Autonomous Surface Ships (MASS). While it is internationally agreed, that MASS must be at least as safe as manned shipping, approaches to implement safe autonomous collision avoidance at sea are widely discussed in the literature. During IMO’s regulatory scoping exercise, it was agreed, that the International Regulations for Preventing Collisions at Sea, 1972 (COLREGs) (IMO (2019)) shall serve as a reference for collision avoidance of MASS (IMO (2021)). Despite COLREGs’ fuzziness with regards to automation applications, striving for COLREGs coverage is remains a challenge when it comes to MASS implementations. This paper’s contribution is reviewing current approaches towards implementing collision avoidance at sea with a focus on assessing COLREGs coverage.

Therefore, it is structured as follows: After an initial introduction into COLREGs and related work in Section 2, Section 3 highlights the research methodology and classification schemes applied. Section 4 contains the list of the investigated literature as well as the main review results per investigated classification category. After the conclusion, a discussion on main gaps with regards to industrialization of the investigated approaches finalizes the paper.

## 2 Background

### 2.1 Collision Avoidance

Regarding collision avoidance of maritime vessels at sea, compliance with decisions made along the prevailing international regulations is mandatory for its acceptability. The regulations are laid out in COLREGs (IMO (2019)), which is subdivided into six parts:

• Part A–General
• Part B–Steering and Sailing
• Part C–Lights and Shapes
• Part D–Sound and Light Signals
• Part E–Exemptions
• Part F–Verification of compliance with the provisions of the Convention

While each vessel participating in the scope of application must comply with all parts of COLREGs during its operation, the most important rules for decision-making during collision avoidance are primarily provided in Part B, hence is in focus of this survey specifically.

### 2.2 Steering and Sailing

Rule 5 to Rule 10 define general guiding principles for all conditions, while Rule 12 to Rule 18 define how collision avoidance shall be executed for a variety of encounter situations in sight of each other. In contrast, Rule 19 defines collision avoidance in conditions of restricted visibility. COLREGs have been written for human operators and interpreted by jurisdictions over time, making a direct transfer into a maritime collision avoidance algorithm non-trivial, however mandatory for later-on industrial operations. The following list gives an at a glance a summary of COLREGs rules most often discussed for collision avoidance algorithms. Anyhow, this selection must not be misinterpreted as a sufficient selection to achieve COLREGs coverage, but shall solely prepare for the following discussions.

• Rule 7–Risk of Collision: All available means shall be used to assess the risk for collision, which specifically exists in case of constant relative bearing.
• Rule 8–Actions to avoid collision: If there is sufficient sea-room, alteration of course alone may be most effective. Reduce speed, stop or reverse only if necessary.
• Rule 13–Overtaking: Any vessel overtaking any other shall keep out of the way of the vessel being overtaken.
• Rule 14–Head-on situation: Each head-on vessel shall alter her course to starboard so that each shall pass on the port side of the other.
• Rule 15–Crossing situation: The vessel which has the other on her own starboard side shall keep out of the way.
• Rule 16–Actions by give-way vessel: Take early and substantial action to keep well clear.
• Rule 17–Actions by stand-on vessel: Keep her course and speed but may take action to avoid collision if the other vessel is not taking appropriate COLREGs compliant action.

### 2.3 Risk Assessment

The risk assessment in accordance with the COLREG rules can be split into four action stages to avoid collision of vessels at sea:

1) No action required–Before risk of collision exists, both ships are free to take any action, which is usually the case whenever the encounter will take place after a long time in the future due to long ranges or slow speeds.
2) Obligation to avoid collision–As soon as the risk of collision begins to arise, the give-way ship is required to take early and substantial action to achieve a safe passage with at a reasonable distance, while the other must keep her course and speed.
3) Last-minute manoeuvre–On appearance, that the give-way ship is not taking appropriate action, the stand-on ship is permitted to take appropriate action to avoid collision by her maneuver alone, however, a power-driven ship must not alter its course to port to avoid collision with another power-driven ship crossing from her port side.
4) Last-second manoeuvre–When a collision is not avoidable by the give-way ship on its own, the stand-on ship is required to take any action that best aids to avoid the collision.

Inertia and intrinsic knowledge of the own ship’s states become progressively more important for autonomous execution of safety-critical maneuvers as needed in stage 3 and stage 4. Hence, the complexity for implementation of the detection and the conduct of the collisions avoidance increases throughout the stages, thus most collision avoidance algorithms govern stage 1 and stage 2 only. Stage 1 and stage 2 can be considered the generic domain of collision avoidance or path planning, respectively, as known from other fields, e.g., robotics.

### 2.4 Related Work

Statheros et al. (2008) is a literature review that is often cited with regards to maritime collision avoidance and COLREGs around the millennia. In recent literature, Salous et al. (2016) conducted a review of 30 approaches for automated collisions avoidance and with a high-level classification of alignment of those approaches to different COLREGs parts. Vagale et al. (2021) contains an overview of several review papers on a variety of MASS topics including a more detailed survey on path planning but without a direct link to COLREGs.

## 3 Methodology

### 3.1 Approach and Objective

The literature review conducted in this survey aims to identify current approaches on collision avoidance at sea, assessing their alignment with COLREGs, and furthermore give an indication about their applicability for MASS as well as its implementation challenges in an industrial context. The literature review is technically based on research utilizing web searches using GoogleScholar and ResearchGate. The search queries were composed of a single or a combination of terms included in the following set:

• COLREG
• Collision Avoidance
• Anti Collision
• Model Predictive Control
• Path Planning

Promising references in the publications found were used to enlarge the observation space even further. The research has been limited to easily accessible or openly available and English-only publications. Furthermore, only papers being published between 2015 and 2020 were considered, since they could not have been in the scope of former reviews discussed above. In terms of automation classes, the focus lays on approaches usable for the *decision and action selection* function of automation, which “*involves selection from among decision alternatives*” (Parasuraman et al. (2000)) in terms of collision avoidance.

At all, 150 publications (133 papers and 17 theses) have been investigated. After an initial investigation of the abstracts and conclusions, out of those 48 publications (including 4 theses) have been identified as appropriate for this survey. Those have either a clear focus on MASS or autonomous systems in general, are intended for use at sea, and have a relevant link to COLREGs.

**FIGURE 1:**
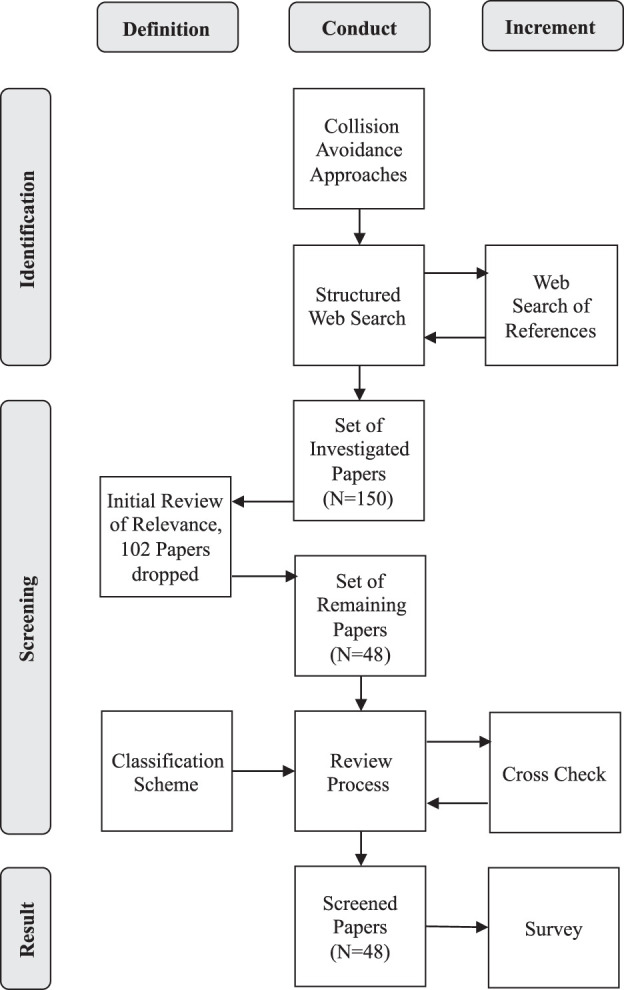
PRISMA flow chart of the review process.

The other papers have primarily being excluded as they did not focus on specific algorithmic decision-making approaches, but, e.g., rather discussed conceptual topics in the area of MASS, or addressed very specific challenges in the field of collision avoidance. Furthermore, the variety of press releases from different industrial actors stating the availability of collision avoidance assistance products seldom contain detailed information about the underlying algorithms. Thus, those kinds of publications have not been investigated. In Figure 1, the review process of this survey is illustrated by a PRISMA flow chart: 1) identification of promising papers via web search, 2) dropping of non-relevant papers in the screening phase, and 3) compiling of results.

### 3.2 Classification Scheme

Each of the 48 remaining publications has been reviewed by an individual and cross-checked by another individual with reasonable knowledge and experience with regard to the scope of this survey.

**FIGURE 2:**
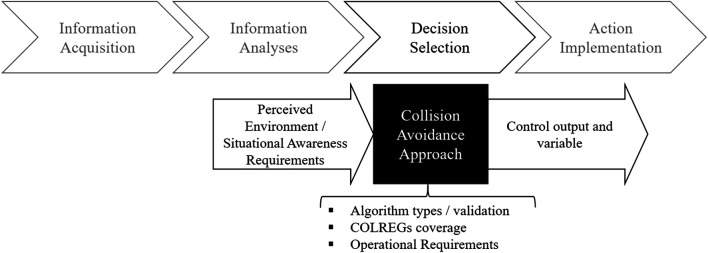
Associations to collision avoidance in terms of automation.

The results of the individual reviews have been condensed in a structured way to a list with predefined multiple-choice categories originated from relevant and important features for collision avoidance approaches in the context of automation as shown in Figure 2, as those seem to exploit the most relevant features for the purpose of this survey, e.g., what kind of sensors were used to perceive the environment, the algorithm classes used, and the area of operation. The following items provide the genuine thoughts for the resultant classification scheme:

• Algorithms and techniques–What is the algorithmic foundation of the proposed approach?
• Algorithm validation–How has the collision avoidance approach being tested?
• Operational requirements–Where (and when) can the approach be applied?
• Control output and variables–How does the collision avoidance mechanism interact with the ship system to really conduct a manoeuvre?
• Situational Awareness’ needs–What are the requirements to observe and perceive the surrounding, i.e., what sensors and data have been utilized?
• COLREGs coverage–Which set of COLREGs rules or types of maritime situations, respectively, is the proposed collision avoidance algorithm able to consider?

## 4 Results

### 4.1 Algorithmic Landscape

Most publications implemented the concept of the Closest Point of Approach (CPA) as a trigger for the activation of collision avoidance or evaluation of the current situation, respectively. Whenever another ship exceeds a predefined or computed threshold in both, time and distance, according to its relative motion, the algorithm steps in and computes an alternative route or signals commands to the helm. This was expected since the CPA is both, well-known and a classical means in nautical navigation to avoid collisions at sea.

The Model Predictive Control (MPC) is also a classical approach to maintain a certain level of predictive control for the behavior of non-linear and complex dynamical systems such as sailing ships with a set of constraints for the actions feasible while conducting actual collision avoidance. The idea of MPC has been used in most publications reviewed in this survey.

Modern techniques like Deep Learning, specifically Reinforcement Learning, have been used rarely for the classical challenge of collision avoidance with respect to COLREGs. Recurrent Neural Networks (RNN), namely Long short-term memory (LSTM) seem to go alongside Deep Learning approaches in a Supervised Learning setup as the driving architecture. However, approaches utilizing Deep Learning, especially in a Reinforcement Learning setup, have been proposed in a minor set of the publications reviewed. Recently, an increasing number of papers have been published utilizing Deep Learning in the context of collision avoidance at sea.

Another algorithm class used frequently is the Artificial Potential Field (APF). An APF creates attractive and repulsive fields on a map or scene. Such interfering fields can be imagined as resultant potential fields exerting forces dependent on the distance to the own ship to guide it on a safe route around static or dynamic obstacles, e.g., other ships for collisions avoidance.

The following list provides an overview of the algorithms and algorithm classes found for collisions avoidance at sea throughout the research for this survey:

• Model Predictive Control (MPC)
• Artificial Potential Field (APF)
• Closest Point of Approach (CPA)
• Deep Reinforcement Learning (DRL) in conjunction with LSTM architectures and PPO optimizers
• Fuzzy Logic
• Genetic Algorithms (GA)

The following algorithms are mostly used for solving the problem of motion and path planning, which can be considered a subproblem of collision avoidance.

• Velocity Obstacle (VO) including optimizations (e.g., Reciprocal VO)
• Optimal Reciprocal Collision Avoidance (ORCA)
• A-star including optimizations (e.g., space-time)
• Rapidly exploring random trees (RRT)

Finally, the activation or evaluation of collision avoidance is often aligned to the sector model of COLREGs Part C–lights and shapes since they provide easy to adapt rules to trigger any collision avoidance algorithm.

### 4.2 Validation Methods

Generically, it must be noted that up to now no standardized or internationally agreed validation method for collision avoidance algorithms exists. However, despite the existence of initial high-level class society guidelines on autonomous navigation as e.g., by DNV (DNVGL (2018)) or Bureau Veritas (Bureau Veritas, 2019), no paper addressed those specifically. Only Hu et al. (2017) mentioned a general link to a class.

**FIGURE 3:**
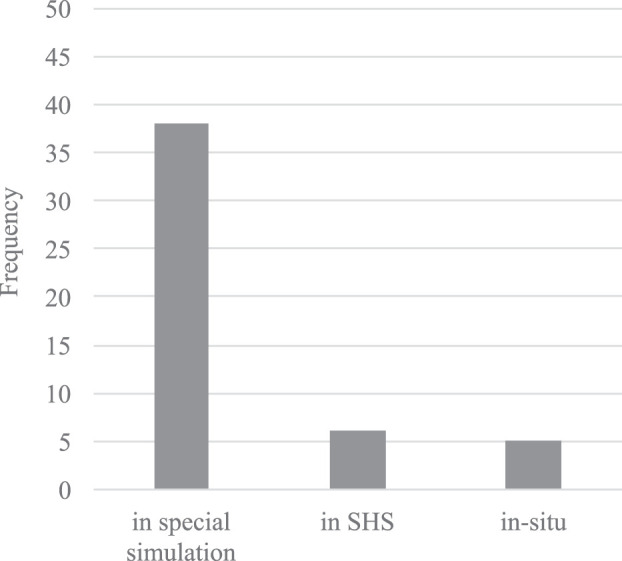
Bar chart of validation platforms for the proposed collision avoidance algorithms.

Figure 3 gives an overview of the generically applied validation environment. Validation of the proposed algorithms–if executed–was mostly done in special simulation environments developed by the respective authors themselves, which are often only described on a high-level basis. Only a small subset has been demonstrated and tested in a commercial ship-handling simulators (Burmeister et al. (2015); Johansen et al. (2016a); Johansen et al. (2016b); Kang et al. (2019); Meyer et al. (2020); Hu et al. (2020)) or even through *in-situ* tests (Mousazadeh et al. (2018); Kufoalor et al. (2019); Liu et al. (2019); Kufoalor et al. (2020)). While the scope of the validation environment in special simulation might differ, in the latter two cases realistic hydro dynamically behavior of the controlled entity can be assumed. However, a certain lack of standardized or at least internationally accepted validation methods for collision avoidance at sea must be acknowledged.

### 4.3 Operational Characteristics

All papers investigated aimed for an IMO MASS Degree One *Seafarers are on board to operate […] Some operations may be automated and at times be unsupervised* or IMO MASS Degree Four *The operating system of the ship is able to make decisions and determine actions by itself* (IMO (2021)). In total, 29 approaches targeted Degree One and 18 approaches even aimed for Degree Four.

**FIGURE 4:**
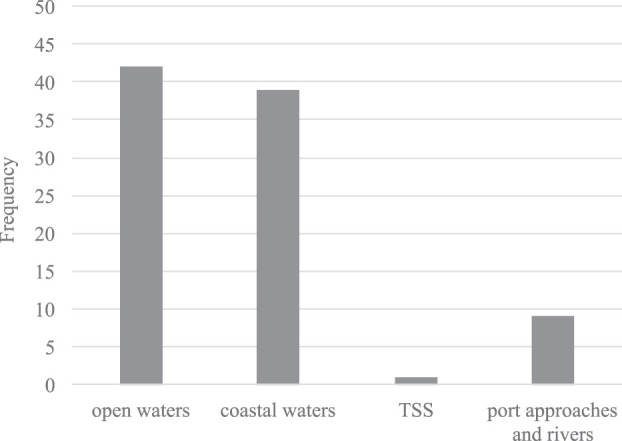
Bar chart of the targeted or inferred area of operation of the proposed approaches.

As shown in Figure 4, nearly all investigated approaches covered collision avoidance in open waters, meaning a freely navigable sea area with other COLREGs vessels present, but without the presence of landmasses or shallow waters. A major subset did also consider the operational area of coastal waters, where fixed non-ship obstacles are taken into account, but the availability of freely navigable sea area can still be assumed. However, only Kufoalor et al. (2020) covered specifically operating in Traffic Seperation Schemes (TSS), which is a likely scenario in coastal waters. Port approaches and rivers, where navigable spaces is restricted to certain lanes, have only been covered by the approaches of Naeem et al. (2016); Tsou (2016); Mei and Arshad (2017); Chen et al. (2018); Mousazadeh et al. (2018); Bevelsborg (2019); Liu et al. (2019); Kang et al. (2019); Zhang X. et al. (2019).

With regards to the maximum number of targets taken into account, only Chiang and Tapia (2018) and Meyer et al. (2020) tested their approaches with more than 10 vessels. 14 papers only tested with single encounters and approximately half of the approaches did not make any specific statement on the number of targets, neither directly nor due to the validation tests described.

### 4.4 Control Output

**FIGURE 5:**
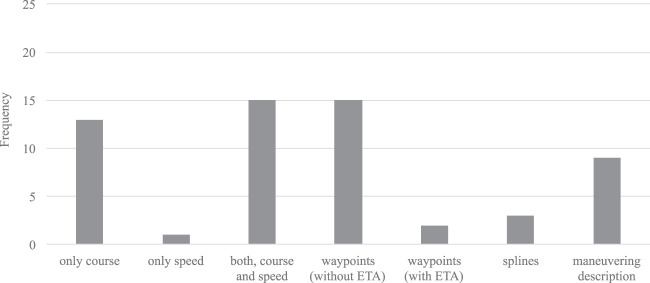
Bar chart of control outputs for the conduct of actions in terms of collision avoidance.

In principle, collision avoidance at sea is a decision-making problem. However, when it comes to defining the *decision* that is made by the approaches, the picture is heterogeneous. As indicated in Figure 5, this paper differentiates in pure COLREGs decisions (course, speed, and a combination thereof), path planning like decisions with waypoints or even ship-specific maneuvering planning. Most approaches tend to purely determine course or course and speed changes, but also collision avoidance waypoint planning is common. In a few cases splines of the recommended route (Wang et al. (2017a); Zhang X. et al. (2019)) or detailed manoeuvre descriptions (Johansen et al. (2016b); Kozynchenko (2018); Eriksen et al. (2019); Kufoalor et al. (2019)) are provided.

How the collision avoidance approach is then linked to the remaining ship system and actuators is not commonly elaborated. Burmeister et al. (2015); Johansen et al. (2016a); Johansen et al. (2016b); Kufoalor et al. (2019) described a direct link between the controller and the onboard auto- or track pilot. Instead, Ning (2018); Eriksen et al. (2019), Beser and Yildirim (2018); Meyer et al. (2020); Ning et al. (2020); Penttinen (2020) assumed direct access to the engine order telegraph (EOT) and rudder control. Burmeister et al. (2015) also described a human readable recommendation system besides the track pilot link. Control semantics used are mainly not specified, but Burmeister et al. (2015) proposed to use the standardized route plan exchange format (RTZ–see IEC (2015), Annex S) in case of waypoint list exchanges as output.

### 4.5 Situational Awareness Requirements

**FIGURE 6:**
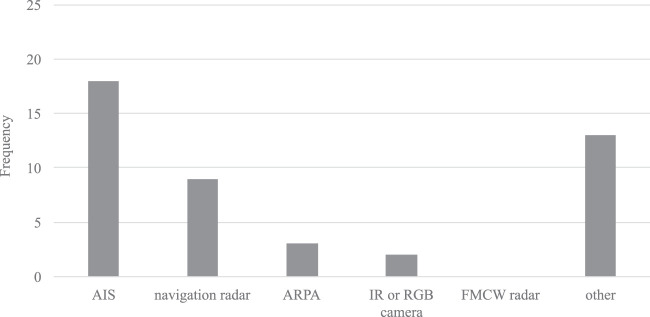
Bar chart of sensor types utilized for perceiving objects and environment.

Decision-making relies on information previously gathering and analyzed (Parasuraman et al. (2000)). This *perceived environment* forms the basis for the collision avoidance functions. If specified, most approaches rely on dynamic data being derived from the Automatic Identification System (AIS) and standard maritime navigation radars (including the Automatic Radar Plotting Aid ARPA). Only Kufoalor et al. (2020) and Mousazadeh et al. (2018) did also refer to camera sensors (see Figure 6).

However, as the decision-making itself should be sensor-agnostic, it is important “*to define a (minimum) data set to be provided by any sensor system enabling the decision-making capabilities of the MASS*’ *navigation system.*”, that should presumably cover traffic object, own ship, and environmental information (Burmeister et al. (2020)). As such an agreed (minimum) data set is not yet in place, a common view on a definition of this perceived environment has been derived from the surveyed approaches along with those three categories.

Traffic object data has been used by all approaches. According to the clustering scheme used in Burmeister and Bruhn (2015), all approaches relied at least on *classified object*, meaning that the object status as *ship* was known and principle position and speed data were available, even though specifically *Rate of Turn* values were not commonly considered. However, only Burmeister et al. (2015); Johansen et al. (2016b); Johansen et al. (2016a); Kufoalor et al. (2018) considered handling of *identified objects*, that included respecting a navigational status provided from a situational awareness system. Kufoalor et al. (2019) is also the only approach identified, that considered sensor uncertainties in obstacle detection by partly working with interval values.

Naturally, dynamic own ship data has also been used by all approaches, even though sometimes the approaches did only rely on relative values and not on a position in a global reference frame, as e.g., in Naeem et al. (2016). Additionally, differentiation between *Speed over Ground* and *Speed through Water* has not been considered in all cases or has not been specified in detail, as e.g., in Hu et al. (2017); Beser and Yildirim (2018); Ning et al. (2020). Indeed, one-third of the approaches relied on ship-specific maneuvering models, requiring more detailed position and velocity data in at least three degrees of freedom (surge, sway, and yaw) to function. These collision avoidance models using ship modeling as a baseline are primarily driven by a research cluster from NTNU in Norway (see Johansen et al. (2016b), Johansen et al. (2016a), Kufoalor et al. (2019), Eriksen et al. (2019), Meyer et al. (2020); Kufoalor et al. (2020)).

**FIGURE 7:**
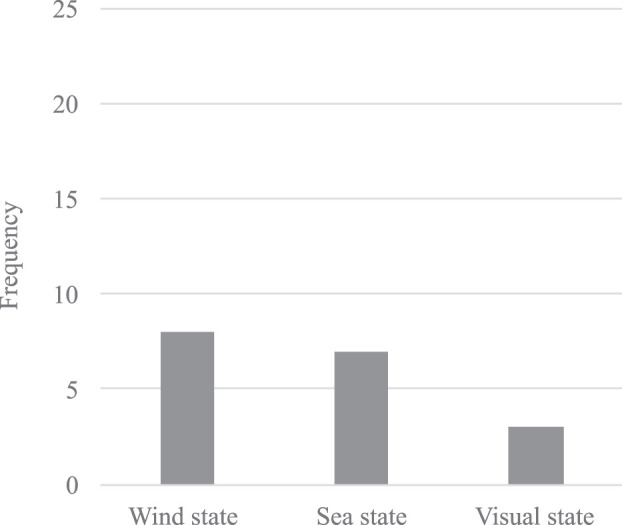
Bar chart of the environmental data used.

Use of environment data was less frequent, as only a subset of less than 20 percent of the investigated papers considered wind and sea state data (see Figure 7). Interestingly, information about the visibility–a piece of the necessary information to determine the COLREGs sections to be used–was only foreseen to be optionally considered in Burmeister et al. (2015) and Hornauer (2016) as well as on a vessel basis in Liu et al. (2019). Despite technical advancements made in recent years, COLREGs still distinguish rules and responsibilities between different visibility states. If COLREGs shall still serve as the backbone and Rule 19 is not changed, this differentiation is necessary, and neglecting this information is not an option. Thus, the operational design domain for most of the investigated algorithms must either be limited to good visibility or handling this information must be included in the algorithms to be fully COLREGs-compliant.

### 4.6 COLREGs Coverage

Besides a wide variety of approaches published on maritime collision avoidance, it must be noted that most approaches in science do often only cover and discuss approaches regarding the basic rules regarding *Overtaking* (R13), *Head-on encounter* (R14) and *Crossing* (R16) as well as the associated rules regarding *Give-way* (R16) and *Stand-on* obligations (R17), and are thus only covering collision avoidance situation in sight of each other. Some papers, i.e., Burmeister et al. (2015); Johansen et al. (2016a); Johansen et al. (2016b); Hu et al. (2017); Eriksen et al. (2019), Eriksen and Breivik (2019); Kufoalor et al. (2019); Kufoalor et al. (2020); Ning et al. (2020); Penttinen (2020) also developed approaches to address the need for an *observable maneuver* (R8).

At the same time, it became clear that nearly no paper provided an approach regarding some typical encounter situations in high-density traffic areas, being operations in *Traffic Separation Schemes* (R9) and *Narrow Channels* (R10). Therefore, most of the approaches are only partly applicable for major shipping lines in coastal waters. With regards to possible COLREGs Part B compliance, an outstanding approach identified is Kufoalor et al. (2020), which is addressing the needs of nearly all COLREGs Part B Rules in a certain way besides *Safe speed* (R6). In line with previous review from Salous et al. (2016), COLREGs Part C and D played no role in the investigated approaches.

A detailed overview about the COLREGs addressed by the respective surveyed papers is given in Table 1, with an “x” marking *addressed* and “/” indicating *partly addressed*. One can observe that the authors do not try to make a juridical assessment if the approach itself is complying with COLREGs, but only highlight if the respective need for a certain rule is generically addressed by the proposed approach.

**TABLE 1:** Papers 1 to 10: COLREGs rules addressed by investigated approaches.

Paper	COLREGs rules addressed													
	5	6	7	8	9	10	12	13	14	15	16	17	18	19
Beser and Yildirim (2018)	-	-	/	-	-	-	-	-	/	/	/	-	-	-
Bevelsborg (2019)	-	-	-	-	-	-	-	x	x	x	x	x	-	-
Burmeister et al. (2015)	-	-	x	x	-	-	-	x	x	x	x	x	x	x
Chen et al. (2018)	-	-	/	-	-	-	-	-	-	-	-	-	-	-
Chiang and Tapia (2018)	-	-	-	-	-	-	-	-	x	x	/	/	-	-
Eriksen et al. (2019)	-	-	x	x	-	-	-	x	x	x	x	x	-	-
Eriksen and Breivik (2019)	-	-	-	x	-	-	-	x	/	/	-	x	-	-
Fang et al. (2018)	-	-	-	/	/	-	-	x	x	x	x	x	-	-
Gao and Shi (2020)	-	-	-	-	-	-	-	x	x	x	-	-	-	-
Guo et al. (2020)	-	-	-	-	-	-	-	x	x	x	x	x	-	-
Hornauer (2016)	-	-	/	/	-	-	-	-	x	x	/	/	-	-
Hu et al. (2017)	-	-	x	x	-	-	-	x	x	x	x	x	-	-
Hu et al. (2020)	-	-	-	-	-	-	-	x	x	x	x	x	-	-
Johansen et al. (2016b)	/	-	x	x	-	-	-	x	x	x	x	-	/	-
Johansen et al. (2016a)	/	-	x	x	-	-	-	x	x	x	x	-	/	-
Kang et al. (2019)	-	-	-	-	-	-	-	-	-	-	-	-	-	-
Kozynchenko (2018)	-	-	-	-	-	-	-	-	-	x	x	x	-	-
Kufoalor et al. (2018)	-	-	-	-	-	-	-	x	x	x	x	x	-	-
Kufoalor et al. (2019)	-	-	x	x	-	-	-	x	x	x	x	x	-	-
Kufoalor et al. (2020)	x	-	x	x	x	x	x	x	x	x	x	x	x	x
Li and Ma (2016)	-	-	-	-	-	-	-	x	x	x	x	x	-	-
Li et al. (2019)	-	-	-	-	-	-	-	x	x	x	-	-	-	-
Liu et al. (2019)	-	-	-	-	-	-	-	-	-	-	-	-	-	-
Lyu and Yin (2019)	-	-	-	-	-	-	-	x	x	x	x	x	-	-
Mei and Arshad (2017)	-	-	-	-	x	-	-	x	x	-	/	-	-	-
Meyer et al. (2020)	-	-	x	/	/	-	-	x	x	-	x	-	/	-
Mousazadeh et al. (2018)	-	-	-	-	-	-	-	-	-	-	-	-	-	-
Naeem et al. (2016)	-	-	-	-	-	-	-	x	x	x	x	x	-	-
Ning (2018)	-	-	-	-	-	-	-	x	x	x	-	-	-	-
Ning et al. (2020)	-	-	x	x	-	-	-	/	x	x	x	/	-	-
Park et al. (2019)	-	-	-	-	-	-	-	x	x	x	x	x	-	-
Pedrielli et al. (2020)	-	-	-	-	-	-	-	-	-	-	-	-	-	-
Penttinen (2020)	-	-	-	x	-	-	-	x	x	x	x	x	-	-
Sawada et al. (2020)	-	-	-	-	-	-	-	/	/	/	-	-	-	-
Szlapczynski et al. (2018)	-	-	-	-	-	-	-	-	x	x	x	-	-	x
Tsou (2016)	-	-	-	-	-	-	-	-	-	-	-	-	-	-
Wang et al. (2017c)	-	-	-	-	-	-	-	x	x	x	-	-	-	-
Wang et al. (2017a)	-	-	-	-	-	-	x	-	-	-	x	x	-	-
Wang et al. (2017b)	-	-	-	-	-	-	-	x	x	x	x	x	-	-
Zaccone et al. (2019)	-	-	-	-	-	-	-	x	x	x	-	-	-	-
Zeng et al. (2019)	-	-	-	-	-	-	-	x	x	x	x	x	-	-
Zhang et al. (2015)	-	-	-	-	-	-	-	-	/	x	x	x	-	-
Zhang et al. (2019b)	-	-	-	-	-	-	-	-	-	-	-	-	-	-
Zhang et al. (2019a)	-	-	-	-	-	-	-	/	/	/	-	-	-	-
Zhao et al. (2016)	-	-	-	-	-	-	-	x	x	x	x	x	-	-
Zhao and Roh (2019)	-	-	-	-	-	-	-	x	x	x	x	x	-	-
Zhao et al. (2019)	-	-	-	-	-	-	-	x	x	x	x	-	-	-
Zheng et al. (2020)	-	-	-	-	-	-	-	x	x	x	x	x	-	-
Total	1	0	9	19	2	1	2	31	34	34	28	24	2	3

## 5 Conclusion

More than 150 publications have been sighted throughout in this survey, and 48 publications including journal articles, conference articles and theses have been evaluated using the described methodology. Hereby, the scientific landscape of collision avoidance at sea can be assessed active during the last 5 years, with most publications proposing optimization of existing algorithms and methods for the special maritime collision avoidance use case. Key characteristics of the landscape are:

• A majority of approaches only addresses Overtaking, Head-on and Crossing encounters, but does not go into the specifics of further COLREGs rules.
• Applicability of the proposed approaches in coastal waters is limited to areas allowing free surface navigation; decision-making in lane-oriented areas like narrow channels and Traffic Separation Schemes is seldom addressed.
• Links to existing onboard systems for input as well as control (as e.g., ECDIS, INS, ENC, Sensors, Track Pilot, DP System) are mostly only briefly discussed.
• Different approaches rely on different information needs from sensor systems, seldom including environmental data.
• Most papers assume a perfect information set as sensors failures are mostly not tackled by the decision-making capabilities.
• Validation of approaches has primarily been executed in stand-alone tests with simulators, seldom in real environment.
• No common testing and validation scheme exists.

In this paper we focused on collision avoidance and its implementation, however, we can not draw a recommendation of appropriate algorithms since neither detailed evaluations nor performance benchmarks regarding the proposed algorithms were highlighted in most of the investigated papers. Moreover, there is no evidence for the existence of an internationally agreed performance benchmark. Specifically for data-driven and trained models with Machine Learning testing the proper function remains challenging especially in the domain of safety-critical applications like collision avoidance. This is due to the usage of sensory data input from the standard equipment onboard and the challenge of operationalization of the fuzziness of COLREGs for autonomous use.

## 6 Discussion

Regarding the applicability of current approaches for MASS in an industrial context, this paper concludes that a gap remains between published research output and industrial implementation needs. This gap can be described along three dimensions:

1. Lacking COLREGs Part B Coverage As COLREGs will remain the backbone of Collision Avoidance at sea (IMO (2021)), approaches aiming to be utilized in MASS’ with higher degrees will need to integrate the whole set of rules within Part B to be applicable. As shown by this review, fully addressing Part B is not common in recent developments. In the industrial context, efforts need to be taken to integrate the currently missing rules, instead of focusing on new approaches only for *Overtaking*, *Head-on*, and *Crossing*. Specifically handling of *traffic separation schemes* and *narrow channels and fairways* is needed.
2. Lacking Perceived Environment Data set DefinitionCollision avoidance approaches do require reliable data sets. In contrast to e.g. car or airplane industry, where sensor and decision logic is typically integrated by the manufacturer and by design in a product, this is not the case in maritime industry. As there are no standardized ships, also their bridges are often individually equipped with different components from different manufacturers. Thus, it is quite likely in the current maritime navigation business, that they might be implemented in a modular way. Most of the investigated approaches do only briefly tackle sensors and uncertainties, as this is part of a separate, but related field of research. The current approaches show certain similarities with regard to data needs but are still varying. However, to fully achieve the possibility to develop sensor-agnostic collision avoidance approaches for MASS, a harmonized or even standardized description of the maritime perceived environment as an interface is necessary to facilitate further development and later-on interoperability in an industrial context (Burmeister et al. (2020)). Besides dynamic sensor data, this might also need an agreement regarding availability and accessibility on further onboard data sets, e.g. *monitored route* and bathymetry data from updated Electronic Nautical Charts. This might also facilitate common test scenario data sets.
3. Lacking Common Test ProceduresCollision Avoidance is safety-critical, thus decision-making approaches in a MASS case bear a great responsibility. Despite this fact, validation and testing in the investigated papers is currently unstructured and not harmonized with a variety of different approaches and data sets, making comparisons and external safety assessment challenging. Thus, common test criteria and data sets must be established to allow for a better comparison between developed approaches and to finally reach the objective to make MASS at least as safe as manned shipping.
As those three dimensions are of special importance for progressing from a research to an industrial application, thorough reporting on those issues is needed in future literature about collision avoidance at sea.

## Data Availability

The original contributions presented in the study are included in the article/supplementary material, further inquiries can be directed to the corresponding author.

## References

[B1] Beser F. Yildirim T. (2018). Colregs Based Path Planning and Bearing Only Obstacle Avoidance for Autonomous Unmanned Surface Vehicles. Proced. Computer Sci. 131, 633–640. 10.1016/j.procs.2018.04.306

[B2] Bevelsborg R. W. O. G. (2019). A Novel Fast Marching Approach for COLREGS Compliant Dynamic Obstacle Avoidance for Unnanned Surface Vehicles. Ph.D. thesis, Available at: http://resolver.tudelft.nl/uuid:3c22ad76-0d80-4d28-88eb-ca1ff2d62b72 .

[B3] Bureau Veritas (2019). Guidelines For Autonomous Shipping (NI641). Available at: https://marine-offshore.bureauveritas.com/ni641-guidelines-autonomous-shipping (Accessed: January 07, 2021).

[B4] Burmeister H.-C. Bruhn W. (2015). “Designing an Autonomous Collision Avoidance Controller Respecting Colreg,” in Maritime-Port Technology and Development : Proceedings of the Conference on Maritime-Port Technology, MTEC 2014, Trondheim, Norway, 27-29 October 2014. Editors Ehlers S. Asbjornslett B. E. Rodseth O. J. Berg T. E. (CRC Press), 83–88.

[B5] Burmeister H.-C. Bruhn W. Walther L. (2015). Interaction of Harsh Weather Operation and Collision Avoidance in Autonomous Navigation. TransNav 9, 31–40. 10.12716/1001.09.01.04

[B6] Burmeister H.-C. Constapel M. Ugé C. Jahn C. (2020). From Sensors to MASS: Digital Representation of the Perceived Environment Enabling Ship Navigation. IOP Conf. Ser. Mater. Sci. Eng. 929, 012028. 10.1088/1757-899x/929/1/012028

[B7] Chen P. Huang Y. Mou J. van Gelder P. H. A. J. M. (2018). Ship Collision Candidate Detection Method: A Velocity Obstacle Approach. Ocean Eng. 170, 186–198. 10.1016/j.oceaneng.2018.10.023

[B8] Chiang H.-T. L. Tapia L. (2018). COLREG-RRT: An RRT-Based COLREGS-Compliant Motion Planner for Surface Vehicle Navigation. IEEE Robot. Autom. Lett. 3, 2024–2031. 10.1109/LRA.2018.2801881

[B10] DNVGL (2018). Class Guideline: Autonomous And Remotely Operated Ships (DNVGL-CG-0264) Available at: https://rules.dnvgl.com/docs/pdf/DNVGL/CG/2018-09/DNVGL-CG-0264.pdf (Accessed January 07, 2021).

[B11] Eriksen B.-O. H. Bitar G. Breivik M. Lekkas A. M. (2019). Hybrid Collision Avoidance for ASVs Compliant with COLREGs Rules 8 and 13-17. Front. Robot. AI 7, 1–18. 10.3389/frobt.2020.00011 PMC7805726 33501180

[B12] Eriksen B.-O. H. Breivik M. (2019). Short-term ASV Collision Avoidance with Static and Moving Obstacles. Mic 40, 177–187. 10.4173/MIC.2019.3.4

[B13] Fang M.-C. Tsai K.-Y. Fang C.-C. (2018). A Simplified Simulation Model of Ship Navigation for Safety and Collision Avoidance in Heavy Traffic Areas. J. Navigation 71, 837–860. 10.1017/S0373463317000923

[B14] Gao M. Shi G.-Y. (2020). Ship-Collision Avoidance Decision-Making Learning of Unmanned Surface Vehicles with Automatic Identification System Data Based on Encoder-Decoder Automatic-Response Neural Networks. Jmse 8, 754–817. 10.3390/jmse8100754

[B15] Guo S. Zhang X. Zheng Y. Du Y. (2020). An Autonomous Path Planning Model for Unmanned Ships Based on Deep Reinforcement Learning. Sensors 20, 426. 10.3390/s20020426 PMC7013856 31940855

[B16] Hornauer S. (2016). Maritime Trajectory Negotiation for N-Vessel Collision Avoidance. carl-von-ossietzky universität oldenburg, 141. Available at: http://oops.uni-oldenburg.de/2861/ .

[B17] Hu L. Naeem W. Rajabally E. Watson G. Mills T. Bhuiyan Z. (2020). A Multiobjective Optimization Approach for COLREGs-Compliant Path Planning of Autonomous Surface Vehicles Verified on Networked Bridge Simulators. IEEE Trans. Intell. Transport. Syst. 21, 1167–1179. 10.1109/tits.2019.2902927

[B18] Hu L. Naeem W. Rajabally E. Watson G. Mills T. Bhuiyan Z. (2017). COLREGs-Compliant Path Planning for Autonomous Surface Vehicles: A Multiobjective Optimization Approach * *The Authors Should like to Thank Innovate UK, grant Reference, TSB 102308, for the Funding of This Project. IFAC-PapersOnLine 50, 13662–13667. 10.1016/j.ifacol.2017.08.2525

[B19] IEC (2015). IEC 61174: Maritime Navigation and Radiocommunication Equipment and Systems – Electronic Chart Display and Information System (ECDIS) – Operational And Performance Requirements, Methods of Testing and Required Test Results. International Electrotechnical Commission. Available at: https://www.vde-verlag.de/iec-normen/222054/iec-61174-2015.html (Accessed January 07, 2021).

[B20] IMO (2019). COLREG - Collision Regulations 1972. London: International Maritime Organisation Available at: https://www.imo.org/en/About/Conventions/Pages/COLREG.aspx (Accessed January 07, 2021).

[B21] IMO (2021). Msc.1/circ.1638 Outcome of the Regulatory Scoping Exercise for the Use of Maritime Autonomous Surface Ships (Mass)

[B23] Johansen T. A. Cristofaro A. Perez T. (2016a). Ship Collision Avoidance Using Scenario-Based Model Predictive Control**This Work Was Supported by the Research Council of Norway, Statoil, DNV GL and Sintef through the Centers of Excellence Funding Scheme, Project Number 223254 - Centre for Autonomous Marine Operations and Systems (NTNU-AMOS), and the Research Council of Norway, DNV GL, Kongsberg Maritime and Maritime Robotics through the MAROFF Knowledge Project 244116 Sensor Fusion and Collision Avoidance for Autonomous Surface Vessels. IFAC-PapersOnLine 49, 14–21. 10.1016/j.ifacol.2016.10.315

[B24] Johansen T. A. Perez T. Cristofaro A. (2016b). Ship Collision Avoidance and COLREGS Compliance Using Simulation-Based Control Behavior Selection with Predictive hazard Assessment. IEEE Trans. Intell. Transport. Syst. 17, 3407–3422. 10.1109/TITS.2016.2551780

[B25] Kang L. Lu Z. Meng Q. Gao S. Wang F. (2019). Maritime Simulator Based Determination of Minimum DCPA and TCPA in Head-On Ship-To-Ship Collision Avoidance in Confined Waters. Transportmetrica A: Transport Sci. 15, 1124–1144. 10.1080/23249935.2019.1567617

[B26] Kozynchenko A. I. Kozynchenko S. A. (2018). Applying the Dynamic Predictive Guidance to Ship Collision Avoidance: Crossing Case Study Simulation. Ocean Eng. 164, 640–649. 10.1016/j.oceaneng.2018.07.012

[B27] Kufoalor D. K. Brekke E. F. Johansen T. A. (2018). “Proactive Collision Avoidance for ASVs Using A Dynamic Reciprocal Velocity Obstacles Method,” in IEEE International Conference on Intelligent Robots and Systems, Madrid, Spain, 2402–2409. 10.1109/iros.2018.8594382

[B28] Kufoalor D. K. M. Johansen T. A. Brekke E. F. Hepsø A. Trnka K. (2020). Autonomous Maritime Collision Avoidance: Field Verification of Autonomous Surface Vehicle Behavior in Challenging Scenarios. J. Field Robotics 37, 387–403. 10.1002/rob.21919

[B29] Kufoalor D. K. M. Wilthil E. Hagen I. B. Brekke E. F. Johansen T. A. (2019). “Autonomous Colregs-Compliant Decision Making Using Maritime Radar Tracking and Model Predictive Control,” in 2019 18th European Control Conference, ECC 2019, Naples, Italy, 2536–2542. 10.23919/ECC.2019.8796273

[B31] Li J. Wang H. Zhao W. Xue Y. (2019). Ship's Trajectory Planning Based on Improved Multiobjective Algorithm for Collision Avoidance. J. Adv. Transportation 2019, 1–12. 10.1155/2019/4068783

[B32] Li W. Ma W. (2016). Simulation on Vessel Intelligent Collision Avoidance Based on Artificial Fish Swarm Algorithm. Polish Maritime Res. 23, 138–143. 10.1515/pomr-2016-0058

[B33] Liu C. Mao Q. Chu X. Xie S. (2019). An Improved A-star Algorithm Considering Water Current, Traffic Separation and Berthing for Vessel Path Planning. Appl. Sci. 9, 1057. 10.3390/app9061057

[B34] Lyu H. Yin Y. (2019). COLREGS-constrained Real-Time Path Planning for Autonomous Ships Using Modified Artificial Potential Fields. J. Navigation 72, 588–608. 10.1017/S0373463318000796

[B35] Mei J. H. Arshad M. R. (2017). “COLREGs Based Navigation of Riverine Autonomous Surface Vehicle,” in USYS 2016 - 2016 IEEE 6th International Conference on Underwater System Technology: Theory and Applications, Penang, Malaysia, IEEE, 145–149. 10.1109/USYS.2016.7893915

[B36] Meyer E. Heiberg A. Rasheed A. San O. (2020). Colreg-Compliant Collision Avoidance for Unmanned Surface Vehicle Using Deep Reinforcement Learning. IEEE Access 8, 165344–165364. 10.1109/access.2020.3022600

[B37] Mousazadeh H. Jafarbiglu H. Abdolmaleki H. Omrani E. Monhaseri F. Abdollahzadeh M.-r. (2018). Developing a Navigation, Guidance and Obstacle Avoidance Algorithm for an Unmanned Surface Vehicle (USV) by Algorithms Fusion. Ocean Eng. 159, 56–65. 10.1016/j.oceaneng.2018.04.018

[B38] Naeem W. Henrique S. C. Hu L. (2016). A Reactive COLREGs-Compliant Navigation Strategy for Autonomous Maritime Navigation. IFAC-PapersOnLine 49, 207–213. 10.1016/j.ifacol.2016.10.344

[B39] Ning J. Chen H. Li T. Li W. Li C. (2020). COLREGs-Compliant Unmanned Surface Vehicles Collision Avoidance Based on Multi-Objective Genetic Algorithm. IEEE Access 8, 190367–190377. 10.1109/access.2020.3030262

[B40] Ning J. (2018). Ships Autopilot Design for Automatic Collision Avoidance Based on Adaptive Neural Networks. Ph.D. thesis World Maritime University (WMU). Available at: https://commons.wmu.se/all_dissertations/627/ .

[B41] Parasuraman R. Sheridan T. B. Wickens C. D. (2000). A Model for Types and Levels of Human Interaction with Automation. IEEE Trans. Syst. Man. Cybern. A. 30, 286–297. 10.1109/3468.844354 11760769

[B42] Park J. Choi J. Choi H. T. (2019). COLREGS‐compliant Path Planning Considering Time‐varying Trajectory Uncertainty of Autonomous Surface Vehicle. Electron. Lett. 55, 222–224. 10.1049/el.2018.6680

[B43] Pedrielli G. Xing Y. Peh J. H. Koh K. W. Ng S. H. (2020). A Real Time Simulation Optimization Framework for Vessel Collision Avoidance and the Case of singapore Strait. IEEE Trans. Intell. Transport. Syst. 21, 1204–1215. 10.1109/TITS.2019.2903824

[B44] Penttinen S. (2020). COLREG Compliant Collision Avoidance Using Reinforcement Learning. Ph.D. thesis. Åbo Akademi University. Available at: https://www.doria.fi/handle/10024/177467 .

[B45] Salous M. Hahn A. Denker C. (2016). “COLREGs-Coverage in Collision Avoidance Approaches: Review and Identification of Solutions,” in 12th International Symposium on Integrated Ship’s Information Systems & Marine Traffic Engineering Conference, Hamburg, Germany, 1–10.

[B46] Sawada R. Sato K. Majima T. (2020). Automatic Ship Collision Avoidance Using Deep Reinforcement Learning with LSTM in Continuous Action Spaces. J. Mar. Sci. Technol. 26, 509–524. 10.1007/s00773-020-00755-0

[B47] Statheros T. Howells G. Maier K. M. (2008). Autonomous Ship Collision Avoidance Navigation Concepts, Technologies and Techniques. J. Navigation 61, 129–142. 10.1017/S037346330700447X

[B48] Szlapczynski R. Krata P. Szlapczynska J. (2018). Ship Domain Applied to Determining Distances for Collision Avoidance Manoeuvres in Give-Way Situations. Ocean Eng. 165, 43–54. 10.1016/j.oceaneng.2018.07.041

[B49] Tsou M.-C. (2016). Multi-target Collision Avoidance Route Planning under an ECDIS Framework. Ocean Eng. 121, 268–278. 10.1016/j.oceaneng.2016.05.040

[B50] Vagale A. Oucheikh R. Bye R. T. Osen O. L. Fossen T. I. (2021). Path Planning and Collision Avoidance for Autonomous Surface Vehicles I: a Review. J. Mar. Sci. Technology (Japan), 2018–2028. 10.1007/s00773-020-00787-6

[B51] Wang T. Yan X. P. Wang Y. Wu Q. (2017b). Ship Domain Model for Multi-Ship Collision Avoidance Decision-Making with COLREGs Based on Artificial Potential Field. TransNav 11, 85–92. 10.12716/1001.11.01.09

[B52] Wang T. Yan X. Wang Y. Wu Q. (2017a). “A Distributed Model Predictive Control Using Virtual Field Force for Multi-Ship Collision Avoidance under COLREGs,” in 2017 4th International Conference on Transportation Information and Safety, ICTIS 2017 - Proceedings, Banff, Canada, 296–305. 10.1109/ICTIS.2017.8047780

[B53] Wang X. Liu Z. Cai Y. (2017c). The Ship Maneuverability Based Collision Avoidance Dynamic Support System in Close-Quarters Situation. Ocean Eng. 146, 486–497. 10.1016/j.oceaneng.2017.08.034

[B54] Zaccone R. Martelli M. Figari M. (2019). “A Colreg-Compliant Ship Collision Avoidance Algorithm,” in 2019 18th European Control Conference, ECC 2019, Naples, Italy, 2530–2535. 10.23919/ECC.2019.8796207

[B55] Zeng Y. Zhang J. Zhang M. Tingwen L. (2019). “Anti-collision Decision Making by Course Alteration and Speed Change under COLREGs,” in ICTIS 2019 - 5th International Conference on Transportation Information and Safety, Liverpool, United Kingdom (IEEE), 25–31. 10.1109/ICTIS.2019.8883588

[B56] Zhang J. Hu Q. Liao B. (2019a). Ship Collision Avoidance Decision Model and Simulation Based on Collision circle. TransNav 13, 325–334. 10.12716/1001.13.02.08

[B57] Zhang J. Zhang D. Yan X. Haugen S. Guedes Soares C. (2015). A Distributed Anti-collision Decision Support Formulation in Multi-Ship Encounter Situations under COLREGs. Ocean Eng. 105, 336–348. 10.1016/j.oceaneng.2015.06.054

[B58] Zhang X. Wang C. Liu Y. Chen X. (2019b). Decision-making for the Autonomous Navigation of Maritime Autonomous Surface Ships Based on Scene Division and Deep Reinforcement Learning. Sensors 19, 4055. 10.3390/s19184055 PMC6767677 31546977

[B59] Zhao L. Roh M.-I. (2019). COLREGs-compliant Multiship Collision Avoidance Based on Deep Reinforcement Learning. Ocean Eng. 191, 106436. 10.1016/j.oceaneng.2019.106436

[B60] Zhao L. Roh M. I. Lee S. J. (2019). Control Method for Path Following and Collision Avoidance of Autonomous Ship Based on Deep Reinforcement Learning. J. Mar. Sci. Technology (Taiwan) 27, 293–310. 10.6119/JMST.201908//27(4).0001

[B61] Zhao Y. Li W. Shi P. (2016). A Real-Time Collision Avoidance Learning System for Unmanned Surface Vessels. Neurocomputing 182, 255–266. 10.1016/j.neucom.2015.12.028

[B62] Zheng M. Xie S. Chu X. Zhu T. Tian G. (2020). Research on Autonomous Collision Avoidance of Merchant Ship Based on Inverse Reinforcement Learning. Int. J. Adv. Robotic Syst. 17, 1–15. 10.1177/1729881420969081

